# Small Intestinal Bacterial Overgrowth and Non-Alcoholic Fatty Liver Disease: What Do We Know in 2023?

**DOI:** 10.3390/nu15061323

**Published:** 2023-03-08

**Authors:** Anna Gudan, Katarzyna Kozłowska-Petriczko, Ewa Wunsch, Tomasz Bodnarczuk, Ewa Stachowska

**Affiliations:** 1 Department of Human Nutrition and Metabolomics, Pomeranian Medical University in Szczecin, 71-460 Szczecin, Poland; 2 Department of Translational Medicine, Pomeranian Medical University in Szczecin, 71-211 Szczecin, Poland; 3 Artimed, Ks. Piotra Ściegiennego 27, 70-354 Szczecin, Poland

**Keywords:** NAFLD, gut–liver axis, SIBO, gut dysbiosis, probiotics, nutrition

## Abstract

Non-alcoholic fatty liver disease (NAFLD) is a chronic liver disease associated with the pathological accumulation of lipids inside hepatocytes. Untreated NAFL can progress to non-alcoholic hepatitis (NASH), followed by fibrosis, cirrhosis, and hepatocellular carcinoma (HCC). The common denominator of the above-mentioned metabolic disorders seems to be insulin resistance, which occurs in NAFLD patients. Obesity is the greatest risk factor for lipid accumulation inside hepatocytes, but a part of the NAFLD patient population has a normal body weight according to the BMI index. Obese people with or without NAFLD have a higher incidence of small intestinal bacterial overgrowth (SIBO), and those suffering from NAFLD show increased intestinal permeability, including a more frequent presence of bacterial overgrowth in the small intestine (SIBO). The health consequences of SIBO are primarily malabsorption disorders (vitamin B12, iron, choline, fats, carbohydrates and proteins) and bile salt deconjugation. Undetected and untreated SIBO may lead to nutrient and/or energy malnutrition, thus directly impairing liver function (e.g., folic acid and choline deficiency). However, whether SIBO contributes to liver dysfunction, decreased intestinal barrier integrity, increased inflammation, endotoxemia and bacterial translocation is not yet clear. In this review, we focus on gut–liver axis and discuss critical points, novel insights and the role of nutrition, lifestyle, pre- and probiotics, medication and supplements in the therapy and prevention of both SIBO and NAFLD.

## 1. Introduction

### 1.1. NAFLD and SIBO—What Do They Have in Common?

Non-alcoholic fatty liver disease (NAFLD), first described in detail in 1980, is now one of the most common diseases in developed countries [1]. It is a chronic liver disease associated with the pathological accumulation of lipids inside hepatocytes. Untreated non-alcoholic fatty liver (NAFL) can progress to non-alcoholic hepatitis (NASH), followed by fibrosis, cirrhosis, and hepatocellular carcinoma (HCC). Factors that contribute to the NAFLD occurrence have been identified, including genetic factors (PNPLA3 rs738409 risk genotype (GG)), obesity, overweight, metabolic syndrome (MS), a highly processed western-type diet rich in sugar and saturated fats as well as insufficient physical activity [1,2,3,4]. The common denominator of the above-mentioned metabolic disorders seems to be insulin resistance (IR), which occurs in NAFLD patients [5,6]. Obesity is the greatest risk factor for lipid accumulation inside hepatocytes, but a part of the NAFLD patient population has a normal body weight according to the BMI index [7]. Moreover, not every person with excess body weight has liver steatosis. Regardless of the potentially diverse etiology of the disease, NAFLD progresses in both slim and overweight people [8]. More and more scientific reports describe NAFLD as the hepatic manifestation of the MS with IR in the background [1]. However, the heterogenicity of the NAFLD pathogenesis is vast, as NAFLD can also be diagnosed in people without MS. Moreover, some people with diagnosed NAFLD may not be at increased risk of cardiovascular disease, and this may be partially mediated via hepatokines (e.g., Fetuin-A, SHBG) [9,10].

The root cause of IR in lean people is still not well understood. Disproportionate body fat distribution (especially visceral adiposity) and adipose tissue dysfunction play a pivotal role in the pathogenesis of IR and NAFLD in lean phenotype [11,12]. Studies show that, at least partially, this phenomenon may also be caused by intestinal inflammation, gut dysbiosis as well as *Helicobacter pylori* infection [13]. Moreover, gastrointestinal motility disorders are much more common in patients with more pro-inflammatory cytokines, especially in diabetic patients [14]. Obese people with or without NAFLD have a higher incidence of small intestinal bacterial overgrowth (SIBO) and, on the other hand, those suffering from NAFLD show increased intestinal permeability and are diagnosed more frequently with SIBO [15,16]. 

SIBO can be caused by gastrointestinal motility disorders (prolonged oro-caecal passage time; OCTT), changes in the anatomy of the gut, or loss of gastric acid secretion (hypochlorhydria) [15]. The disorder usually causes malnutrition and weight loss due to malabsorption. In a healthy small intestine, the growth of native bacterial flora in the jejunum ranges from 10^2^ to10^5^ colony forming units per milliliter (CFU/mL) [16]. Aerobic and anaerobic organisms multiply when the small intestine is stagnant for some reason and when there is dysfunction of the migrating myoelectric complex (MMC). As aerobic and anaerobic microbiota proliferate, they can interfere with absorption, compete for nutrients, and produce substances that trigger clinical symptoms [15]. Under physiological conditions, the maintenance of homeostasis in the small intestine is possible due to the correct production of gastric acid, the protective secretory function of IgA (sIgA), proper cleansing of the intestines with MMC or the production of an appropriate amount of bacteriostatic bile [16]. Clinical symptoms vary depending on the cause of SIBO, but the most common symptoms can be distinguished: abdominal pain, flatulence, gas, diarrhea, constipation [17]. The most common causes of SIBO are impaired anatomy (e.g., bariatric surgery, diverticulosis, stenosis), motor disorders (e.g., diabetic enteropathy, scleroderma, pseudo-enteric obstruction), and various other conditions in which the small intestine becomes diseased and stagnant, including, e.g., post-infection states. Recently, more and more attention has been focused on the cause of chronic use of drugs from the group of proton pump inhibitors (PPIs) [17,18]. The health consequences of SIBO are primarily malabsorption disorders (in particular vitamin B12, iron, choline, fats, carbohydrates and proteins) [17,19] and bile salt deconjugation [20]. However, whether SIBO directly contributes to decreased intestinal barrier integrity, increased inflammation, endotoxemia and bacterial translocation is not yet clear [21,22,23]. It has been shown that intestinal dysbiosis, endotoxemia and bacterial translocation may contribute to inflammation and IR [22,24,25]. This directly seems to disrupt the functioning of the gut–liver axis, which may influence the incidence and progression of NAFLD [26].

Under physiological conditions, the gut microbiome is kept in strict homeostasis [16]. The intestinal microorganisms produce short-chain fatty acids (SCFA), namely acetate, butyrate and propionate [27]. SCFA influence the hepatic metabolism by directly altering the liver metabolism and by influencing epigenetic mechanisms. The bile acids (BA) produced in the liver are also processed by the gut microbiota and released [20]. Patients with diagnosed NAFLD show a significant qualitative and quantitative disturbance in the intestinal microbiome, in particular the growth of Gram-negative bacteria typical of SIBO [17]. This bacterial overgrowth leads to a reduction in bacterial diversity and the production of pro-inflammatory molecules such as Lipopolysaccharides (LPS), ethanol, trimethylamine (TMA)and bacterial genetic material [23,28]. These molecules reach liver cells through the portal vein and may ansilate inflammation and both liver steatosis and fibrosis, thus potentially accelerating NAFLD progression [28,29]. On the other hand, it is known that both intestinal insulin/IGF1 signaling through FoxO1 [30,31] and hyperglycemia regulate epithelial integrity and drive intestinal barrier dysfunction [32]. Impaired insulin signaling and hyperglycemia are diagnosed among patients with NAFLD [33,34]; therefore, intestinal barrier disruption and intestinal permeability may equally result from these mechanisms.

### 1.2. The Aim of the Study

In this review, we present the details of the functioning of the gut–liver axis, particularly explaining the mechanisms and the correlation between the occurrence of SIBO and NAFLD. In addition, we discuss critical points, novel insights and the role of nutrition, lifestyle, pre- and probiotics, medication and supplements in the therapy and prevention of both.

## 2. Non-Alcoholic Fatty Liver Disease 

### 2.1. Diagnosis

The first choice diagnostic method of hepatic steatosis as recommended by the European Association for the Study of the Liver (EASL) is the ultrasound [35]. It is a safe, simple, inexpensive, and widely available method. Although subjective, this method can diagnose moderate to severe hepatic steatosis (>20–30% liver tissue) with high sensitivity (84.8%) and specificity (93.6%) [36]. Newer quantitative ultrasound methods offer higher diagnostic reliability and reproducibility than semi-quantitative methods. They use additional data based on acoustic parameters of liver tissue. Among the best studied are ultrasound attenuation parameters. Widely validated and increasingly used for non-invasive assessment of liver steatosis, controlled attenuation parameter (CAP) is obtained simultaneously with vibration-controlled transient elastography (VCTE) measurement of liver stiffness available on the Fibroscan^®^ device (Echosens, Paris, France). In a meta-analysis involving 2735 patients, the high diagnostic value of this method against biopsy for the diagnosis of hepatic steatosis was proven (AUROC 0.82) [37]. Despite its many advantages, CAP’s limitations include the determination of the optimal cutoff point for each degree of hepatic steatosis and the impact of probe choice on the test result. According to EASL guidelines, CAP assessment should be compared with ultrasound, which despite its limitations, remains the most widely used tool for detecting hepatic steatosis [38]. Several ultrasound machine manufacturers have patented software to quantify ultrasound beam attenuation similar to the CAP described earlier. The tools developed to date that have been integrated into ultrasound machines are attenuation imaging (ATI) and ultrasound-guided attenuation parameter (UGAP) [39]. There are also several methods for quantifying hepatic steatosis based on magnetic resonance (MR) techniques. The two main MR-based methods include MR spectroscopy (MRS) and the MR-Proton Density Fat Fraction (MRI-PDFF). These techniques have the highest diagnostic accuracy, but due to the cost and limited availability of these methods, they are not used in routine diagnosis [40].

### 2.2. Treatment 

The goal of NAFLD treatment is not only to reduce hepatic steatosis, but also to treat the components of the metabolic syndrome. In patients with simple hepatic steatosis (without accompanying inflammation and fibrosis of the organ), it is recommended to limit pharmacotherapy to comorbidities (obesity, type 2 diabetes, hyperlipidemia or hypertension), good control of which is also a prerequisite for successful treatment of hepatic steatosis [41]. Sustained lifestyle modification, consisting of dietary changes and increased moderate physical activity, is fundamental to the successful treatment of NAFLD-induced hepatic steatosis [41,42]. In addition, it is of great supportive importance in the treatment of comorbid components of the metabolic syndrome. The patient should be aware that lifestyle modification in the treatment of simple hepatic steatosis is clearly superior to pharmacotherapy and in most cases achieves therapeutic success. The goal of non-pharmacological management is gradual loss of excess body weight [43]. NAFLD-targeted pharmacotherapy should be implemented only in patients with histologically confirmed NASH or liver fibrosis [41]. 

As the association between NAFLD and SIBO has been already observed, there are some trials on the potential benefit of probiotics or synbiotics therapy by NAFLD patients [44,45,46]. Significant therapeutic effect was proven in fatty liver mice models. Moreover, some human trials showed that probiotics may improve transaminase levels and hepatic steatosis. Undoubtedly, further studies are needed to clearly assess the efficacy of probiotics in the management of NAFLD [46]. 

## 3. Small Intestinal Bacterial Overgrowth 

### 3.1. About SIBO

Small intestinal bacterial overgrowth (SIBO) is a heterogeneous disorder characterized by excessive growth of selected microorganisms in the small intestine [47]. More and more studies indicate that the overgrowth of small intestine bacteria is a common clinical problem [48]. SIBO may be asymptomatic or one may have symptoms that indicate a different disease. 

Thus far, there are four known types of SIBO:

1. Hydrogen SIBO. Usually associated with gastrointestinal symptoms such as bloating, excess gas, abdominal pain, diarrhea and weight loss [49].
2. Methane SIBO, more often called IMO (intestinal methanogen overgrowth). IMO is a methanogen proliferation syndrome in the small intestine. The characteristic symptoms include abdominal pain, nausea, difficulty passing bowel movements and changing bowel habits. In this case, too much methane production is caused not by bacterial overgrowth but by the type of archaea, mainly *Methanobrevibacter smithii.* Due to the overgrowth of these archaea, the use of the term IMO seems more appropriate than “SIBO” or “Methane-SIBO”. Although methanogens are found in the small intestine, people who test positive for exhaled methane also have elevated levels of methanogens in their stools, suggesting that they may be present throughout the digestive tract [48,50].
3. Hydrogen-methane SIBO (mixed). Characteristic of this hypertrophy is the occurrence of both diarrhea and constipation. The longer the imbalance in the intestinal microbiome lasts, the greater the likelihood of intestinal dysbiosis [51,52].
4. Hydrogen-sulfide SIBO. The characteristic symptoms are gas and stools with the smell of hydrogen sulfide, halitosis, chronic fatigue, headaches, and fibromyalgia. Symptoms worsen with sulfur-rich foods found in diets or supplements [52]. Moreover, patients in hydrogen–methane tests at each stage of the study report low levels of methane and hydrogen despite a number of symptoms [51,53].

### 3.2. Diagnosis

The gold standard for the diagnosis of bacterial overgrowth in the small intestine is the microbiological examination of the content of the small intestine and the determination of the number of bacetria equal or above 10^5^ cfu/mL of intestinal content. Due to the high cost of the examination and its invasive nature, this method is not used in the diagnosis of SIBO [48]. For this purpose, respiratory tests are used to diagnose several gastroenterological conditions, including SIBO, poor digestion of carbohydrates, dysfunction, or changes in the ileocecal passage [50]. The tests are performed using a hydrogen analyzer or a hydrogen–methane analyzer. The performed tests are called hydrogen and hydrogen–methane tests, respectively [51]. The premise of breathing tests is that human cells are unable to produce hydrogen and methane. Consequently, if these gases can be detected in samples of exhaled air, they must represent a different source, such as fermentation of carbohydrates by microbes in the gut, their subsequent absorption into the bloodstream and their excretion through the lungs [54]. Detection of SIBO and its type (methane, hydrogen, or methane–hydrogen) involves the patient consuming a solution of lactulose (10 g) or glucose (75 g) dissolved in 250 mL of water. Then, the exhaled air is analyzed by a suitable device. Measurement of the metabolites in the expired air is repeated every 20 min for a period of 3 h [50].

According to the American College of Gastroenterology (ACG) guidelines, an increase of ≥20 ppm from the baseline value up to 90 min of the test is a positive hydrogen test result [51,54]. We consider a positive methane test result to be an increase of ≥10 ppm at any time during the breath test [51].

### 3.3. Treatment

Treatment of SIBO is determined individually by the attending physician. In this case, antibiotic therapy turns out to be effective, depending on the type of SIBO [15]. The attending physician does not always decide to administer the antibiotic treatment. Everything depends on the presence of comorbidities, what is the primary cause of SIBO, organ damage and symptoms with which the patient consults the doctor. In addition, the doctor may consider adding additional substances, such as prokinetics or probiotics (specific strains). In 2020, the American College of Gastroenterology (ACG) developed guidelines for the use of antibiotics, as presented below in the Table 1 [51].

The most common antibiotic is rifaximin [55]. It is an oral antibiotic with a broad spectrum of antibacterial activity against Gram-positive and Gram-negative aerobic and anaerobic bacteria. The addition of a pyridoimidazole ring to the rifampicin molecule makes rifaximin insoluble in water and thus poorly absorbed in the gastrointestinal tract [55]. It works locally, not systemically. It is considered an eubiotic, i.e., a type of antibacterial drug that, when introduced into the body, changes the composition of the intestinal microflora. However, it does not affect the microbiome of the large intestine and has a positive effect on the intestinal barrier integrity. In addition, rifaximin reduces the level of blood ammonia. A recent study by Zhang et al. in patients with cirrhosis of the liver showed that rifaximin at a dose of 200 mg three times daily for one week was effective in reducing the symptoms of SIBO along with hepatic encephalopathy [56]. The concentration of ammonia in the patient’s blood also decreased significantly (from 56.1 to 39.1 μmol/L, *p* < 0.01) [57]. The number of side effects is low; hence, rifaximin is considered a safe, well-researched and highly effective drug. This type of antibiotic is used mostly for hydrogen SIBO (the diarrheal form) [17,55].

Neomycin belongs to the group of aminoglycoside antibiotics, which is another antibiotic used in the treatment of SIBO [58]. It has a strong bactericidal effect, especially against Gram-negative bacteria and some Gram-positive bacteria [59]. Neomycin is highly effective for the treatment of IMO, in particular in combination with rifaximine [60]. Two studies investigated the efficacy of neomycin in constipated patients. In the first study by Pimentel M. [61], 84 patients with IBS and IMO received neomycin 500 mg twice daily for 10 days. In 20% of the patients, the methane level dropped below 3 ppm compared to the patients who received the placebo. There, the effectiveness was 1%. The second study was a retrospective review of lactulose breath tests in 74 IMO patients [62]. In this case, the subjects received either neomycin alone (500 mg twice daily), rifaximin only (400 mg three times daily), or both antibiotics for 10 days. The reduction of the methane level below 3 ppm in repeated breath tests in people treated with neomycin alone was 33%, in people treated with rifaximine 28%, and in people treated with both antibiotics, the effectiveness was 87% [51]. Neomycin, like rifaximin, is slightly absorbed in the gastrointestinal tract, acting locally and not systemically; therefore, it is often used in both IMO and mixed SIBO, in combination with rifaximine [60].

Metronidazole, a chemotherapeutic agent, has strong bactericidal properties against anaerobic bacteria, including *Helicobacter pylori* and *Clostridium difficile* [63]. It is also applicable in the case of infections with protozoa, giardiasis (*Giardia lamblia*) as well as methanogenic archaea [64]. Contrary to rifaximine, metronidazole quickly enters the gastrointestinal tract directly through the mucosa, which carries the risk of mycosis after antibiotic treatment [65]. This drug is ineffective against relatively anaerobic and aerobic bacteria. This type of antibiotic often combined with rifaximine is used in IMO (constipation predominant) or mixed SIBO (SIBO/IMO, diarrheal-constipated form).

During antibiotic treatment, probiotics with the non-pathogenic yeast *Saccharomyces boulardi CNCM I-745* can be used. Probiotics with *Lactobacillus* strains are generally not recommended, as they may worsen symptoms [66].

## 4. Gut–Liver Axis

### 4.1. Gut–Liver Axis in Health and Disease

The gut mucosal barrier is a functional unit composed of the gut microbiota, the mucus layer, which contains antimicrobial products (e.g., defensin) and the secretory IgA, the epithelium built of specialized cells connected by intercellular junctions, and finally the gut-associated lymphoid tissue. The mucus secreted by intestinal goblet cells prevents the direct contact between intestinal microorganisms and the epithelium. In this way, the intestinal barrier plays a role of both a physical and immunological barrier that prevents bacterial adhesion and controls paracellular trafficking. In addition to the mucus and the epithelial layer, the gut–vascular barrier prevents translocation of bacteria and directly into the portal circulation [67]. The close anatomical localization with the portal circulation system and biliary tree links the liver and the gut to configure bidirectional crosstalk. A wide variety of bacterial metabolites, food antigens, or xenobiotics reach the liver through the intestinal barrier with the portal blood, exposing the liver to continuous immunogenic stimuli. On the other side, liver-derived molecules such as cytokines, bile acids and hormones reach the gut with the bile, shaping the gut immune system and regulating the intestinal microbiome [68]. In this way, in health, the liver continuously maintains the balance between immune defense and tolerance. Furthermore, the disruption of the gut barrier under unfavorable conditions such as alcohol consumption, inflammation and dysbiosis leads to intestinal permeability and subsequent increased hepatic exposure to a range of proinflammatory molecules. This pathogenic pathway activates the immune system and triggers hepatic and finally systemic inflammation [26,69].

### 4.2. Intestinal Permeability and NAFLD

In NAFLD, associations between dysbiosis, barrier damage, hepatic inflammation and metabolic abnormalities under high-fat diet conditions have been confirmed both in animal model studies and in humans. In a mice model, Rahman et al. [70] showed that loss of intestinal barrier integrity promotes severe steatohepatitis on a diet high in saturated fat, fructose, and cholesterol. These data were confirmed in a study in humans. Miele L. et al. [15] showed that in patients with NAFLD, intestinal permeability measured by the urinary excretion of 51Cr-ethylene diamine tetraacetate (51Cr-EDTA) is increased in comparison to healthy controls. In the same study, duodenal expression of zona occludens-1 in intestinal biopsy specimens reflecting tight junction integrity was altered in patients with NAFLD as compared to healthy volunteers [28]. These results suggest that increased intestinal permeability has an important role in the pathogenesis of hepatic steatosis.

### 4.3. Pathogen-Associated Molecular Patterns and NAFLD

The liver is a “first pass” organ exposed to the highest concentration of microbe-derived products such as trimethylamine, secondary bile acids, short-chain fatty acids and ethanol. These molecules constitute pathogen-associated molecular patterns (PAMPs) and cause vulnerable effects on the liver, particularly if preconditioned by steatosis. Intestinal permeability facilitates the portal influx of PAMPS, worsening hepatic inflammation and metabolic abnormalities. The pathogenetic role of microbe-derived metabolites is well established in animal model studies, showing that hepatic steatosis, inflammation and fibrosis are attenuated in TLR-4- and TLR-9-deficient mice on a high-fat or choline-deficient diet [71,72]. Moreover, in a mice model of hepatic steatosis, changes in gut microbiota are associated with exacerbated hepatic steatosis and inflammation through portal influx of TLR4 and TLR9 agonists, leading to enhanced hepatic tumor-necrosis factor (TNF)-α expression that drives NASH progression [73]. In addition, in humans, PAMPS have been shown to play a pathogenic role in NAFLD development and progression. Alterations in intestinal microbiota in NAFLD patients have been linked to increased serum concentrations of pathogenic gut microbiome-derived products, ex. LPS, endotoxin, ethanol, branched-chain and aromatic amino acid metabolism products [74,75]. The pathogenic role of microbiome-derived metabolites is further demonstrated by the fact that transplantation of fecal microbiota from human donors with hepatic steatosis triggered steatosis in recipient mice.

### 4.4. Bile Acid Metabolism and NAFLD

Bile acids (BAs) are steroid molecules derived from cholesterol precursors. Primary bile acids, mainly cholic and chenodeoxycholic acids, are conjugated with glycine or taurine in the liver, and then are incorporated into bile and stored in the gallbladder. In the postprandial period, bile is released from the gallbladder into the duodenum. In the intestine, bile salts are involved in the formation of micelles, digestion and absorption of fats and fat-soluble vitamins. In addition, they play an important role in the formation and functioning of the intestinal microbiome [76]. Bile acids may have antimicrobial activity, which may have a preventive effect on SIBO and gut dysbiosis. Most of the cholic and chenodeoxycholic acid pools are absorbed in the ileum. The remaining amount reaches the large intestine, where it is metabolized by the microbes living there [77]. As a result, secondary bile acids, lithocholic and deoxycholic acids, are formed, which by passive diffusion enter the portal circulation and are transported to the liver. BA are ligands of the FXR receptor (farnesoid X receptor) located in the intestine and liver, involved in the regulation of bile acid synthesis and maintaining homeostasis of glucose and lipid metabolism [78].

There are several representative examples of FXR targets relevant to the pathophysiology of NAFLD, including SREBP1c (de novo lipogenesis), PPARα (fatty acid uptake, binding and oxidation), PEPCK (gluconeogenesis and glyceroneogenesis) and NF-κB (e.g., LPS-mediated inflammation) [76]. In an animal model, FXR-null mice have been shown to develop dyslipidemia and hepatic steatosis, and they exhibit a proatherogenic lipoprotein profile with markedly elevated serum and hepatic cholesterol and triglycerides [79]. Activation of FXR blocks hepatic de novo lipogenesis and stimulates fatty acid β-oxidation, thus limiting intrahepatic lipid accumulation and hepatic steatosis [80,81]. 

The gut microbiome, through specific enzymatic actions (such as deconjugation, dehydroxylation, oxidation, and epimerization), is a critical modulator of BA pool size and composition, and can significantly modify both chemical and signaling properties of the BA [20]. Thus, gut microbiome-targeted therapeutic interventions in NAFLD/NASH may, at least in part, work by inducing changes in host BA profiles. Nutraceutical (diet, probiotics, prebiotics, synbiotics), pharmaceutical (antibiotics, e.g., rifaximine) or surgical (e.g., bariatric surgery) interventions can modify NAFLD phenotype [77], through changes in BA composition that affect signaling through FXR and possibly other BA receptors [20,82,83]. 

### 4.5. Clinical Implications 

**Figure 1:**
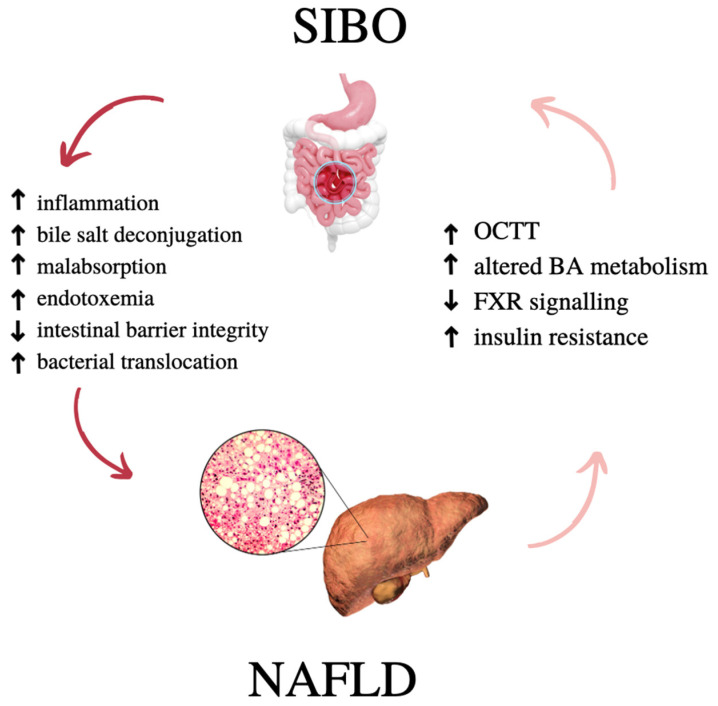
Possible SIBO and NAFLD interactions. OCTT—oro-ceacal transit time; BA—bile acids; FXR—farnesoid X receptor.

Growing evidence from experimental and translational studies support the associations between SIBO and NAFLD. In patients with NAFLD, an increased prevalence of SIBO is often observed, which correlates with the severity of steatosis and hepatic inflammation [67,68]. The crosslink between these two conditions underlines the fact that both NAFLD and SIBO are associated with similar proinflammatory signaling pathways (Figure 1).

As disruption of the gut–liver axis contributes to the pathogenesis of NAFLD, the modulation of its components, especially microbiome composition and the gut–liver signaling pathway, has potential therapeutic implications. These strategies have shown promising results in preclinical studies; however, evidence in human clinical trials is limited thus far. In this review, we present emerging therapeutic approaches targeting the gut–liver axis in NAFLD and SIBO. 

## 5. Gut Microbiota Changes in NAFLD and SIBO

The composition of the gut microbiota and the metabolome produced by microorganisms is one of the most important variables determining human health. Population-based studies comparing people with traditional, low-urbanized lifestyles (e.g., residents of Africa) and developed countries leave no illusions—exposure to antimicrobial agents (in Western life style) negatively affects the diversity of the gut microbiome (reduces the abundance of Lactobacillus, Bacteroides, Prevotella, Desulfovibrio and Oxalobacter in the gut [84]). Significantly the composition, diversity and richness of the gut microbiota determine the efficiency of energy acquisition from food, and the reduction in microorganism diversity is associated with an increase in the incidence of chronic metabolic diseases including NAFLD [85]. A growing body of evidence suggests that altered gut microbiota (described as dysbiosis) may be a crucial factor in the pathophysiology of NAFLD. Abnormalities in liver function associated with dysbiosis are prompted not only by the gut bacteria but also by fungi, viruses, and other microbes [86]. Alterations in the composition of the microbiota in NAFLD and NASH are shown in the Table 2 below [87].

The gut microbiota may be involved in the pathogenesis of NAFLD, via several factors [87]:

(a) Body weight—an increase in body weight contributes to a decrease in the diversity of the gut microbiota [92];
(b) Anatomical and functional changes in the intestinal barrier (defined as the immune barrier, the intestinal vascular barrier, and the hepatic barrier). Intestinal dysbiosis, intensifying the translocation of bacteria through the portal vein to the liver (endotoxemia), enhances inflammatory responses in the liver [93],
(c) Specific patterns pro-inflammatory compounds of the intestinal microbiome—PAMPs and MAMPs such as LPS, peptidoglycans and lipopeptides, microbial DNA, circulating proinflammatory cytokines (IL-1, IL-6, INF-γ, and TNF-α) may contribute to the inflammatory response and fibrosis in patients with NAFLD [87,94];
(d) Could influence the metabolic and inflammatory state of the liver through the release of anti-inflammatory compounds (short chain fatty acids-SCFA) that bind to G protein-coupled receptors (GPCRs) induce hepatic lipids and glucose homeostasis, the regulation of intestinal integrity and intrinsic immune defenses [95].

## 6. Pro-, Pre-, and Symbiotic Therapy in NAFLD and SIBO 

Probiotics and synbiotics appear to improve intestinal barrier function in patients with NAFLD. In meta-analyses published to date, intake of probiotics resulted in minor improvements in several metabolic risk factors in subjects with NAFLD [96], e.g., probiotics/synbiotics were associated with improvement in hepatic steatosis, as graded by ultrasound reduction, in alanine aminotransferase activity, liver stiffness [97] as well as for aspartate transaminase (AST) and total cholesterol (TC) [98]. A recent meta-analysis published in 2021 concluded that probiotic therapy can be used as an effective method to improve liver function and lower blood lipid levels in patients with NAFLD [98].

Improvements were mostly observed with Bifidobacteria (*B. breve, B. longum*), *Streptococcus salivarius* subsp. *thermophilus* and Lactobacilli (*L. acidophilus*, *L. casei*, *L. delbrueckii*) [96].

Fungal products might also be developed as therapies although the potential health benefits, or probiotic effects of some fungal species are known but have yet to be fully explored [86]. Only *Aureobasidium pullulans* obtained from administrated black yeast prevented the develop of fatty liver in mice on high-fat diets [99].

We have only one meta-analysis that confirms the utility of probiotic therapy in patients with SIBO. The results of the meta-analysis indicated that they helped to achieve a higher decontamination rate, reduced hydrogen concentration, reduced abdominal pain, and did not significantly reduce the daily frequency of bowel movements [100]. 

Improvements were observed with Lactobacilli (*L. rhamnosus*, *L. pentosus*, *L. plantarum*, and *L. delbrueckii* subsp. *delbruecki*) and Bifidobacteria (*B. longum*, *B. breve*).

## 7. Nutrition—What Is beyond the Obvious? 

### 7.1. Current State of Knowledge—Diet for SIBO and NAFLD

A healthy diet and increased physical activity are considered the most important part of NAFLD therapy [101]. Nevertheless, the nutrition guidelines for NAFLD patients are not consistent and specific. Scientific associations (ESPEN, APASL, AASLD) emphasize the importance of losing weight using a low-calorie diet (500–1000 kcal/day deficit), as well as the importance of physical activity in reducing IR; however, there are no detailed guidelines in the form of type of diet and exercise [102,103,104]. Most scientific societies indicate the Mediterranean diet as the most beneficial model of nutrition for NAFLD patients, which is confirmed by numerous scientific studies [102,105]. At the same time, these scientific societies indicate that there is insufficient scientific evidence for considering macronutrient distribution as significant. 

Currently, there is a lack of scientific evidence considering a dietary regimen for patients with SIBO [18]. One of the possible routes of nutritional therapy is the low FODMAPs diet (LFD) [106], originally dedicated to patients with irritable bowel syndrome (IBS) [17]. It is estimated that up to 70% of IBS patients may have SIBO as a comorbid condition [15]. There is still lacking data to conclude what is the best dietary intervention for patients with SIBO. Thus far, the most important method of treating SIBO is drug therapy with simultaneous elimination of the causative agent. It is also not known how the low FODMAPs diet affects the parameters of hepatic steatosis in NAFLD patients with coexisting SIBO. Below we present a comparison of chosen innovative diets in Table 3.

### 7.2. Mediterranean Diet

Scientific research shows that the Mediterranean diet (MEDDiet) significantly prevents cardiovascular and the majority of metabolic diseases [107,108]. Typical features of the Mediterranean diet are consumption of olive oil, fruit, vegetables, grains (mainly whole grains), legumes and nuts. Fish, white meat, eggs, and fermented dairy products (cheese and yogurt) are also included, but in moderate amounts. Very small amounts of red meat and high-sugar and processed foods are allowed [108]. Fat consumption accounts for about 40% of daily calories, with an emphasis on monounsaturated fatty acids (MUFA), mostly derived from olive oil (25% of calories). In contrast, saturated fatty acids (SFA) account for less than 8% of fat consumed daily [108,109,110]. The MEDDiet is abundant in omega-3 fatty acids from fish and other seafood and plant sources, and is characterized by low omega-6/omega-3 ratio (2:1–1:1 compared to 14:1 and in western countries) [111]. High intake of dietary fiber and antioxidant compounds shows synergistic beneficial anti-inflammatory effects, especially within the microscopic intestinal barrier and the gut microbiome [112,113]. The Mediterranean diet has in general a strong anti-inflammatory effect [114]. The dominant anti-inflammatory ingredients in this diet are omega-3 fatty acids, olive oil and phenolic compounds, as well as fiber and a low degree of food processing [112,114,115]. 

Components of the Mediterranean diet, mainly polyphenols and fiber, are inversely correlated with the incidence, severity and progression of NAFLD [116]. Overall, studies have shown that adherence to the MEDDiet reduces hepatic steatosis [117]. In addition, the MEDDiet may even be associated with a reduced risk of hepatocellular carcinoma (HCC) or death from liver disease. Meir A.Y et al. in the DIRECT-PLUS clinical trial [118] investigated the Mediterranean diet (MEDDiet) and the green Mediterranean diet (Green MEDDiet) for the effect on hepatic steatosis in NAFLD patients (n-294). The GreenMEDDiet group additionally consumed green tea (3–4 servings/day), *Wolffia globosa* aquatic plant strain (100 g/day frozen cubes) and green shakes with an additional portion of polyphenols in the amount of +1240 mg/day. In this RCT, both MEDDiet and reduced calorie Green-MEDDiet were shown to reduce body weight. Importantly, however, despite similar moderate weight loss in both groups, the green-MEDDiet group achieved almost double the percentage loss of intra-hepatocyte fat content (IHF%) (−38.9%), compared to the MEDDiet (−19.6%; *p* = 0.035) and the general principles of healthy eating (HDG) group (−12.2% proportional; *p* < 0.001). Greater loss of IHF% was independently associated with increased consumption of *Wolffia globosa* and walnuts, decreased consumption of red and processed meat, increased serum folate, changes in microbiome composition (*beta* diversity), and specific bacteria (*p* < 0.05 for all) [118].

### 7.3. Low FODMAPs Diet

The low FODMAPs (LFD) diet is an elimination protocol based on a temporary limitation of the consumption of strongly fermenting substances such as: fructooligosaccharides, oligosaccharides, disaccharides, monosaccharides and polyols [119]. Limiting the consumption of these ingredients significantly reduces fermentation in the intestine, reduces the inflow of water to the intestine and reduces gas production. Altogether, it reduces the sensation of pain, flatulence, diarrhea, while improving the quality of life of patients [23]. The diet is divided into three successive stages: I—elimination (4–8 weeks), II—reintroduction (6–10 weeks) and III—stabilization and expansion (4 weeks and more). LFD should be carried out under the supervision of a qualified dietitian, and elimination and a scanty diet should not be used longer than indicated. Special emphasis should be placed on expanding the diet in the last phase of the protocol [120]. The influence of the low FODMAPs diet on the composition and function of the microbiome is not indifferent. LFD has been shown to significantly reduce the quantitative composition, and in particular, the number of beneficial microorganisms such as *Akkermansia mucinifila*, *Clostridium cluster IV*, *Propionibacteriaceae* and *Bifidobacterium* is reduced [121]. The effectiveness of LFD in SIBO diet therapy seems to be directly dependent on the initial quantitative and qualitative composition of the patients’ gut microbiome. Initially, the respondents showed greater saccharolytic activity of the microbiota as opposed to the non-respondents [122]. The optimal solution may be the use of the complete low FODMAPs diet protocol followed by extension to the Mediterranean diet [123]. It is still not known how LFD affects liver function in patients with NAFLD, and further studies are needed.

### 7.4. Ketogenic Diet

A ketogenic diet (KD) is any diet that puts the body into a state of physiological ketosis. A common feature of all ketogenic diets is a very low supply of carbohydrates, most often oscillating around 5–10% of the total energy supply [124]. Such a low consumption of carbohydrates causes a cascade of physiological reactions related to, among others, increased secretion of glucagon, cortisol, norepinephrine and growth hormone and decreased secretion of insulin and insulin-like growth factor [125]. Lower plasma insulin concentration and progressive depletion of glycogen stores over time lead to the activation of fat reserves and an increase in hepatic ketogenesis to provide cells with an alternative source of energy to glucose [126]. Complete depletion of glycogen reserves and carbohydrate restriction result in the preferential utilization of fatty acids and ketone bodies by peripheral tissues, a state typical of nutritional ketosis [126,127]. Fat energy substrates can have two origins: endogenous (from tissue fat) and exogenous (from food fat) [128]. 

Although the ketogenic diet places great emphasis on adequately low carbohydrate intake, the sources of proteins and fats that replace carbohydrates are usually neglected [129]. Most often, the dietary sources are rich in saturated fatty acids (SFA). It is well known that higher SFA intake is positively correlated with the occurrence and progression of IR, and this is directly related to the occurrence and progression of NAFLD [130,131]. NAFLD patients have an unfavorably high serum triacylglycerol (TAG) level associated with de novo lipogenesis [1]. The accumulating TAGs in serum come mainly from de novo lipogenesis (mainly from glucose), the lipolysis of the adipose tissue and free fatty acids from the daily diet. It should be noted that the level of TAGs generated in the liver from de novo lipogenesis is five times higher in NAFLD patients compared to healthy controls. The probable mechanism responsible for this is the strong hepatic and peripheral IR in these patients. As de novo lipogneesis is largely mediated by circulating blood glucose, a ketogenic diet may be considered a nutritional model for NAFLD patients in the future [132]. However, the reduction of hepatic steatosis mostly takes place when an energy deficit in the diet is ensured; thus, there emerges a problem with lean NAFLD patients [133]. KD is quite effective for weight management and may have additional benefits such as prevention of muscle loss and appetite control [134]. Ketone bodies, mainly beta-hydroxybutyric acid (BHB) produced on a ketogenic diet, have appetite-suppressant properties [135]. This feature may be beneficial in the context of reducing the daily consumption of calories, thus generating an energy deficit, which has a beneficial effect on the reduction of hepatic steatosis [1]. Moreover, BHB, in addition to its inhibitory effect on appetite, has anti-inflammatory properties by directly reducing NLRP3 inflammasome-mediated interleukin (IL)-1β and IL18 production in human monocytes together with lowering of IL1β secretion [135,136]. These are the key inflammatory cytokine related to obesity and IR [6]. In a mouse model, activation of the inflammatory pathway of NLRP3 has been shown to play a key role in inducing liver fibrosis [137]. BHB has anti-inflammatory properties by binding to the HCA2 receptor; thus, it may exert a beneficial effect [135,136].

Holmer M. et al. conducted a randomized controlled trial (RCT) that included 74 patients with NAFLD. Patients went on 12 weeks of treatment with either a low-carbohydrate ketogenic diet or intermittent fasting, or general lifestyle advice from a hepatologist (control group) [138]. Participants in the KD group experienced a greater weight loss and intrahepatic fat content reduction, although the energy deficit was smaller [138]. Unfortunately, in this study, changes in liver fibrosis were not demonstrated. In the study from Cunha G. M. et al., 20 patients on VLCKD (intervention arm) and 19 patients on standard low-calorie diet (control arm) were evaluated at baseline and after 2 months of intervention [139]. In the KD group, there was a statistically significant (*p* < 0.001) greater body weight reduction (−9.59 ± 2.87%) in the VLCKD group in comparison to the low-calorie group (−1.87 ± 2.4%) [139]. Mean reductions in visceral adipose tissue (VAT) were −32.0 cm^2^ for the KD group and −12.58 cm^2^ for the low-calorie group (*p* < 0.05). Reductions in liver fat fraction were significantly more pronounced in the intervention arm than in the control arm (4.77 vs. 0.79%; *p* < 0.005). This study shows that weight loss and rapid mobilization of liver fat demonstrated with a ketogenic diet may be an effective alternative for the dietary management of NAFLD. However, there is still a concern in terms of dietary quality of the ketogenic diet. The ketogenic diet is commonly rich in SFA; thus, it may not be appropriate dietary advice. In response, scientists have set out to create a ketogenic diet model that most closely resembles the established Mediterranean diet model—the Mediterranean ketogenic diet [140].

The Mediterranean ketogenic diet is a relatively new nutritional model based on the principles of carbohydrate restriction [140,141]. Unlike the classic ketogenic diet, its Mediterranean variant is rich in dietary fiber, olive oil, vegetable protein, plant polyphenols, antioxidants and omega-3 fish fats, seeds, nuts and non-industrial vegetable fats [140,142]. This composition of the diet is widely recognized as the most beneficial for patients with MS or cardiovascular disease [141]. Moreover, these nutrients have strong preventive properties against many civilization diseases, including obesity and cancer [108]. Dietary fiber, along with plant polyphenols, is the most important component that causes beneficial changes in the functioning of the intestinal microbiome [112]. It is speculated, that low supply of dietary fiber and plant polyphenols in a classic ketogenic diet may adversely affect the composition and function of the intestinal microbiome, thus adversely affecting the functioning of the liver–gut axis and impeding liver regeneration. A prospective study by Pérez-Guisado J. et al. was carried out during a general medicine consultation in 14 overweight male subjects with MS and NAFLD [143]. All participants were prescribed a Mediterranean ketogenic diet in the Spanish variant. At the end of the study, there was a statistically significant (*p* < 0.001) reduction in body mass (109.79 kg vs. 95.86 kg) and LDL cholesterol (123.43 mg/dL vs. 100.35 mg/dL). There were significant changes in ALT (71.92 U/L vs. 37.07 U/L), AST (47.71 U/L to 29.57 U/L) and steatosis degree (complete NAFL regression (21.4%); overall steatosis reduction (92.86%)). Moreover, in this study, all the parameters studied that were associated with MS were improved: BMI, waist circumference, fasting, TAG, HDLc, systolic blood and diastolic blood. After the intervention, all the patients were free of MS characteristics, and 100% of them had normal TAG and HDL-C levels. However, 100% of the participants still were characterized by a BMI of >30 kg/m^2^ [143]. 

### 7.5. Intermittent Fasting 

**Figure 2:**
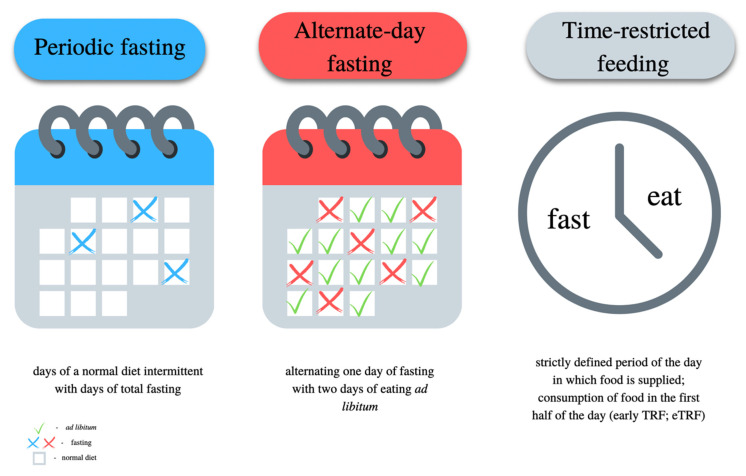
Characteristics of different types of fasting.

Intermittent fasting (IF) has recently gained a lot of attention due to its possible effectiveness in the process of weight reduction [144]. IF is characterized by alternating periods of fasting and feeding. During fasting periods, one must not eat any energetic ingredients—only water, coffee or tea is permitted. Intermittent fasting may be the basis of a personalized diet, which considers not only the proper caloric value and distribution of macronutrients, but also the proper planning of meals daily. Fasting changes the metabolism of hepatocytes into preferential fat oxidation and the formation of ketone bodies, which may contribute to the reduction of hepatic steatosis [145]. There are many types of IF, the most important and the most frequently used of them are presented in Figure 2.

During fasting, hormonal signaling through insulin and the pathways associated with it are reduced. This in turn enhances the action of the FOXO proteins, the FOXO1 protein, which synergistically with TORC2 increases the transcription of the gluconeogenesis and FOXO3 genes involved in longevity. FOXO proteins are important regulatory factors in the metabolism of carbohydrates and lipids, and their activity depends on insulin; hence, they are expressed mainly in insulin-dependent tissues, e.g., liver, muscles or adipose tissue [145]. They undergo numerous post-translational modifications. They are deactivated because of phosphorylation with PKB/Akt, while they are deacetylated with the participation of sirtuins, which activates FOXO proteins and allows them to be moved to the interior of the cell nucleus, where they fulfill their epigenetic functions. FOXO1 proteins increase the expression of genes that promote mitochondrial fatty acid oxidation and reduce the expression of genes that promote glucose utilization [146]. PPAR-a transcription is inhibited, but PPAR-a expression is increased, indicating increased fatty acid oxidation [145,147]. Activation of PPAR-a stimulates the expression of genes responsible for fatty acid oxidation in both mitochondria and peroxisomes, the synthesis of ketone bodies and general fatty acid transformations. The potentiation of the action of PPAR-a is observed during the ketogenic diet, caloric restriction, and starvation. The major inhibitor of PPAR-a is mTOR and the pathways associated with it [148].

An umbrella meta-analysis conducted by Patikorn Ch et al. indicates a possible beneficial effect of intermittent fasting (IF) in overweight, obese, NAFLD or healthy adults compared to a regular diet [149]. In these individuals, IF has been shown to positively influence BMI, body weight, fat mass, triglycerides, LDL-C, total cholesterol, fasting plasma glucose, fasting insulin, HOMA-IR, and systolic and diastolic blood pressure. These indicators are the very determinants of health and disease progression in NAFLD patients. A systematic review by Różański G et al. has confirmed the previous results [150]. The studies included in the systematic review show a significant reduction in waist circumference (100.2 ± 11.0 vs. 99.3 ± 10.9 cm, *p* < 0.001) [151], fat content in men (29.8 ± 6.6% vs. 29.0 ± 6.6%; *p* < 0.001) and women (41.9 ± 6.2% vs. 41.4 ± 6.1%, *p* = 0.03). However, the results of the studies are not consistent, which may result from the different methodologies of these works. One study showed no significant effect of IF on the change in plasma glucose concentration; however, other researchers such as Arabi et al. [152] and Aliasghari et al. [151] observed a significant increase in fasting glucose (85.5 vs. 133.6 mg/dL, *p* < 0.001 in men; 100 vs. 120.2 mg/dL, *p* < 0.001 in women; and 94.0 ± 8.0 vs. 92.0 ± 7.8 mg/dL, *p* < 0.001, respectively). Inconsistencies concern the effect of IF on ALT. Some researchers have shown an increase in ALT after IF, while others have observed a decrease in ALT [150]. 

Studies conducted in people with pre-diabetes, hypertension and MS indicate a beneficial effect of eTRF on metabolic parameters (fasting glucose, fasting insulin, lipid profile, oxidative stress, hypertension, postprandial insulin) [153,154], even without total body fat mass reduction [155]. At the same time, no undesirable side effects of eTRF were observed. However, it is not known how exactly eTRF affects the parameters of steatosis and the progression of NAFLD, although in light of currently known mechanisms and scientific evidence to date, it may be a good therapeutic solution to consider, in combination with the Mediterranean diet model and daily physical activity, especially strength training.

## 8. Conclusions

In summary, the gut–liver axis exists and plays a pivotal role in the homeostasis of human metabolic health. Different molecules, including LPS and PAMPs that come from the intestines, are subjected to the first-pass effect in the liver, consequently impacting liver metabolism and predisposing it toward NAFLD. Intestinal permeability is often observed in patients with NAFLD. Notably, the loss of intestinal barrier integrity promotes severe steatohepatitis on a diet high in saturated fat, fructose, and cholesterol. Gut microbiome and mycobiome dysbiosis can lead to hepatic steatosis, inflammation and NAFLD progression. Selected types of probiotics and synbiotics seem to improve gut–liver function in NAFLD patients, especially those containing *Bifidobacterium breve*, *B. longum*, *Streptococcus salivarius* subsp. *thermophilus* and *Lactobacillus acidophilus*, *L. casei*, *L. delbrueckii*. SIBO is increasingly observed in patients with NAFLD. Untreated SIBO can lead to significant malnutrition (vitamin B12, choline and protein deficiencies), subsequently worsening hepatocyte function and increasing intrahepatic lipid accumulation. Drugs and antibiotics used in SIBO therapy (including rifaximin) seem to improve liver parameters in people with NAFLD. The NAFLD diet therapy includes the introduction of physical activity along with a Mediterranean diet containing dietary fiber, prebiotics, fermented foods, polyphenols and the predominance of monounsaturated fatty acids derived from olive oil. This type of diet is considered to evoke a beneficial effect on liver metabolism, MS, as well as on gut microbiome composition and function. However, people with both SIBO and NAFLD seem to require different diet and drug therapy. To date, no consensus has been established on a specific dietary regimen for SIBO management. The most studied model of nutrition thus far seems to be the low FODMAPs diet, which is low in fiber and low in prebiotics, in contrast to the Mediterranean diet, which is highly recommended for NAFLD patients. Thus, patients with NAFLD may suffer from SIBO, and those diagnosed with both SIBO and NAFLD may require different dietary and pharmacological management.

## Figures and Tables

**Table 1:** American College of Gastroenterology (ACG) developed guidelines for the use of antibiotics [51].

Suggested Antibiotics for the Treatment of Small Intestinal Bacterial Overgrowth	
Drug	Dosage
Rifaximin	550 mg three times per day; 61–78% efficacy
Amoxicillin/clavulanic acid	875 mg twice daily; 50% efficacy
Ciprofloxacin	500 mg twice daily; 43–100% efficacy
Doxycycline	100 mg one-two times daily (efficacy not defined)
Metronidazole	250 mg three times per day; 43–87% efficacy
Neomycin	500 mg twice daily; 33–55% efficacy
Norfloxacin	400 mg once daily; 30–100% efficacy
Tetracycline	250 mg once daily; 87.5% efficacy
Trimethoprim-sulfamethoxazole	160 mg/80 mg twice daily; 95% efficacy

**Table 2:** Gut microbiome and mycobiome changes in NAFLD and NASH.

	NAFLD	NASH	Function Features [85]
Increased in NAFLD Bacteria Fungi	Phylum: *Proteobacteria* [88] Family: *Lactobacillaceae* [89] Genus: *Bacteroides* [90] *Ruminococcus* [90] *Lactobacillus* [89] Species: *E. coli* [88], *Klebsiella pneumoniae* *Streptococcus anginosus* *Veillonella atypica* *Talaromyces*, *Paraphaeosphaeria*, *Lycoperdon*, *Curvularia*, *Phialemoniopsis*, *Paraboeremia*, *Sarcinomyces*, *Cladophialophora*, *Sordaria*	*Ruminococcus*, *Blautia*, *Dorea* [91] *C. albicans*, *Mucor* sp., *Cyberlindnera jadinii*, *Penicillium* sp., unknown *Pleosporales*, *Babjeviella inositovora* and *Candida argentea*	Oxidative damage γ-Aminobutyric acid biosynthesis Dentrification Ethanol production Lipopolysaccharide and peptidoglycan biosynthesis Branched-chain amino acid (BCAAs) and aromatic amino acid (AAA) biosynthesis
Decreased in NAFLD Bacteria Fungi	Phylum: *Actinobacteria* [91], *Bacteroidetes* [91], *Firmicutes* Genus: *Oscillobacter*, *Prevotella* [91], *Ruminococcus* [90], *Coprococcus*, Species: *Faecalibacterium prausnitzii* [89] *Coprococcus comes* *Leptosphaeria*, *Pseudopithomyces*, *Fusicolla*	*Bacteroidetes* [91]	Haem biosynthesis

**Table 3:** Characteristics of different types of diets.

Diet	Ketosis	Kcal per Day	% Macronutrients in Total Calories per Day							
			*CHO*	*Source*	*Protein*	*Source*	*Fats*	*Source*	*Fiber*	*Source*
**Mediterranean diet**	No	Individual, but >1000 kcal per day	>50% energy	whole grain, vegetables, seeds, nuts, starch, fruits	15–20% energy	lean meat, fish, eggs, legumes, lean dairy products, fermented dairy products, seafood, tofu	20–35% energy	olive o oil, fish oil, nuts, seeds, unrefined vegetable oils	30–40 g/day	all vegetables and fruits, nuts, seeds, whole grain, resistant starch, cruciferous, green leafy vegetables, fermented foods, all legumes
**Low FODMAPs diet**	No	Individual, but >1000 kcal per day	>40% energy	low FODMAPSs: whole grain, rice, oats, vegetables, seeds, nuts, fruits	15–20% energy	lean meat, fish, eggs, legumes, lean dairy products, fermented dairy products, seafood, tofu	20–35% energy	olive o oil, fish oil, nuts, seeds, unrefined vegetable oils	Varies, but mostly <25 g/day	all except for fructooligosaccharides, oligosaccharides, disaccharides, monosaccharides and polyols
**Standard ketogenic diet**	Yes	Individual, but >1000 kcal per day	20–30 g/day	some vegetable, some fruits, nuts, seeds	15–20% energy	fatty meat, fatty dairy products, eggs, giblets, fish, seafood, sausages	>60–70% energy	butter, lard, animal fats, nuts, seeds, coconut oil, some MCT oil, little or no vegetable fats	<25 g/day	some vegetables and fruits low in carbohydrates, nuts, seeds, cruciferous and green leafy vegetables, fermented foods
**VLCKD**	Yes	<800 kcal/day	20 g/day	little vegetable, some fruits, nuts, seeds	15% energy	fatty meat, fatty dairy products, eggs, giblets, fish, seafood, sausages	>70% energy	butter, lard, animal fats, nuts, seeds, coconut oil, some MCT oil, little or no vegetable fats	<25 g/day	some vegetables and fruits low in carbohydrates, nuts, seeds, cruciferous and green leafy vegetables, fermented foods
**Mediterranean ketogenic diet**	Yes	Individual, but >1000 kcal per day	50–80 g/day	vegetables, seeds, nuts, fruits	15–20% energy	lean meat, fish, eggs, legumes, lean dairy products, fermented dairy products, seafood, tofu	>50% energy	olive o oil, fish oil, nuts, seeds, unrefined vegetable oils, MCT oil	30–40 g/day	most vegetables and fruits low in carbohydrates, nuts, seeds, cruciferous and green leafy vegetables, fermented foods, some legumes
**Intermittent fasting**	Spontaneous	Individual, but usually >1000 kcal per day	Varies							

## Data Availability

Not applicable.
